# Seroprevalence and risk factors of African horse sickness in equines in central Gondar, Ethiopia

**DOI:** 10.1007/s11259-026-11388-w

**Published:** 2026-07-08

**Authors:** Abebech Atinafu Yehulashet, Fentahun Wondmnew, Anmaw Shite Abat, Enyiew Alemnew Alamerew, Mastewal Birhan, Firdyawukal Abuhay Tafere, Birhan Anagaw Malede, Andebet Sechu, Amsalu Chanie Mersha, Elias Melkamu Tsehay, Abraham Belete Temesgen

**Affiliations:** 1 Department of Veterinary Pharmacy, College of Veterinary Medicine and Animal Sciences, University of Gondar, P.O. Box: 196, Gondar, Ethiopia; 2 Department of Veterinary Pathobiology, College of Veterinary Medicine and Animal Sciences, University of Gondar, Gondar, Ethiopia; 3 Amhara Agricultural Research Institute, Debre Birhan Agricultural Research Centre, Debre Birhan, Ethiopia; 4 Department of Serology, Animal Health Institute, Sebeta, Ethiopia; 5 Department of Veterinary Epidemiology and Public Health, College of Veterinary Medicine and Animal Sciences, University of Gondar, Gondar, Ethiopia

**Keywords:** African horse sickness, AHSV, BELISA, Equine, Seroprevalence

## Abstract

African horse sickness is a severe vector-borne viral disease of equines, associated with high mortality and substantial economic losses. In Ethiopia, where equines are essential for transport and agriculture, the disease remains endemic, yet epidemiological data are limited in many areas. This study aimed to estimate the seroprevalence of African horse sickness virus (AHSV) and describe variation in seropositivity across selected factors in equines in the Central Gondar Zone, Ethiopia. A cross-sectional study was conducted from December 2024 to August 2025, during which 384 serum samples were collected and tested using a blocking enzyme-linked immunosorbent assay (bELISA). Of these, 310 were seropositive, giving an overall seroprevalence of 80.7% (95% CI: 76.4–84.6%). Seroprevalence was slightly higher in East Dembia (81.3%) than in West Dembia (79.9%). Univariable logistic regression showed no statistically significant associations between seropositivity and the evaluated variables (*p* > 0.05). Although not statistically significant, higher odds were observed in donkeys and horses compared to mules, in older animals, and under sheltered management conditions. These findings indicate widespread exposure to AHSV in the study area. However, results should be interpreted cautiously due to the descriptive nature of the analysis. Further studies incorporating larger sample sizes, multivariable approaches, and complementary diagnostic methods are recommended to better understand infection dynamics. Strengthened vaccination, improved management practices, and effective vector control remain essential for reducing disease impact.

## Introduction

African horse sickness (AHS) is a highly fatal, vector-borne viral disease of equines caused by African horse sickness virus (AHSV), a double-stranded RNA virus belonging to the genus *Orbivirus* within the family *Reoviridae* (Azeez 2017). Due to its transboundary potential and severe socioeconomic impact on equine-dependent communities, AHS is listed by the World Organisation for Animal Health (WOAH) as a notifiable disease (Bitew et al. 2011).

Among equine species, horses are the most susceptible, with mortality rates reaching up to 95% during outbreaks (Zeleke et al. 2005). Mules exhibit intermediate susceptibility, with reported mortality rates of approximately 50%, while donkeys and zebras typically develop milder or subclinical infections (Rodríguez et al. 2020; Wassie and Temesgen 2025). Clinically, AHS manifests in four classical forms: pulmonary, cardiac, mixed, and horse sickness fever. The mixed form, which is most frequently observed, is often fatal, whereas horse sickness fever is generally mild and occurs primarily in donkeys and zebras (Ndebé et al. 2022).

Transmission of AHSV occurs predominantly through *Culicoides* biting midges, particularly *Culicoides imicola* and *Culicoides bolitinos*, although other arthropods, including mosquitoes and ticks, have occasionally been implicated (Oura et al. 2012). Disease outbreaks typically coincide with late summer and early autumn, corresponding to peaks in vector density (Ndebé et al. 2022). In insect vectors, the virus persists by evading immune defenses, thereby facilitating efficient transmission. In vertebrate hosts, AHSV preferentially infects vascular endothelial cells, leading to increased vascular permeability, edema, and hemorrhage (Rodríguez et al. 2020).

Diagnosis of AHS is challenging owing to its wide range of clinical presentations, which vary from acute pulmonary and cardiac forms to mild or subclinical infections (Skowronek et al. 1995). The pulmonary form is peracute and usually fatal, whereas the cardiac form is characterized by edema of the head, neck, and supraorbital regions. The mixed form combines features of both pulmonary and cardiac disease. Protective immunity against AHSV is largely serotype-specific, complicating vaccine development and disease control efforts in endemic regions (Ndebé et al. 2022).

Ethiopia possesses the largest equine population in Africa, estimated at approximately 5.42 million donkeys, 1.78 million horses, and 373,000 mules, accounting for 6.9% of the global and 42.4% of the African equine population (Getaw and Dejene 2017). Equines play a crucial role in rural livelihoods by supporting transportation, agricultural activities, and the movement of goods (Tilahun et al. 2012). Despite their economic and social importance, equine health has historically received limited attention, and infectious diseases such as AHS continue to compromise productivity and animal welfare (Getaw and Dejene 2017).

Recurring AHS outbreaks have been documented across multiple regions of Ethiopia, including the Central, Southern, Western, and Northern parts of the country (Aklilu et al. 2014). Notably, the 2002–2003 outbreaks resulted in substantial losses, particularly among donkeys, a species previously regarded as relatively resistant to severe disease (Karamalla et al. 2018). These recurrent epidemics highlight the need for strengthened surveillance systems, improved diagnostic capacity, and systematic epidemiological assessments to better understand disease distribution and risk factors (Nidra et al. 2024; Mohammad 2025). Although vaccination programs are implemented in some areas, continued outbreaks suggest challenges related to vaccine coverage, efficacy, and possible circulation of diverse viral strains (Apostolopoulos et al. 2024; Kamel et al. 2025).

Despite the recognized risk, comprehensive epidemiological data on AHS remain scarce in high-priority areas such as the Central Gondar Zone. Weak surveillance infrastructure and inconsistent vaccination practices continue to hinder effective disease control, allowing AHS to persist and threaten equine health and rural livelihoods. Therefore, this study aimed to estimate the seroprevalence of AHSV among equines in selected districts of the Central Gondar Zone and to describe variations in seropositivity across selected risk factor categories, thereby generating evidence to support improved control and prevention strategies.

## Materials and methods

### Study area

**Fig. 1:**
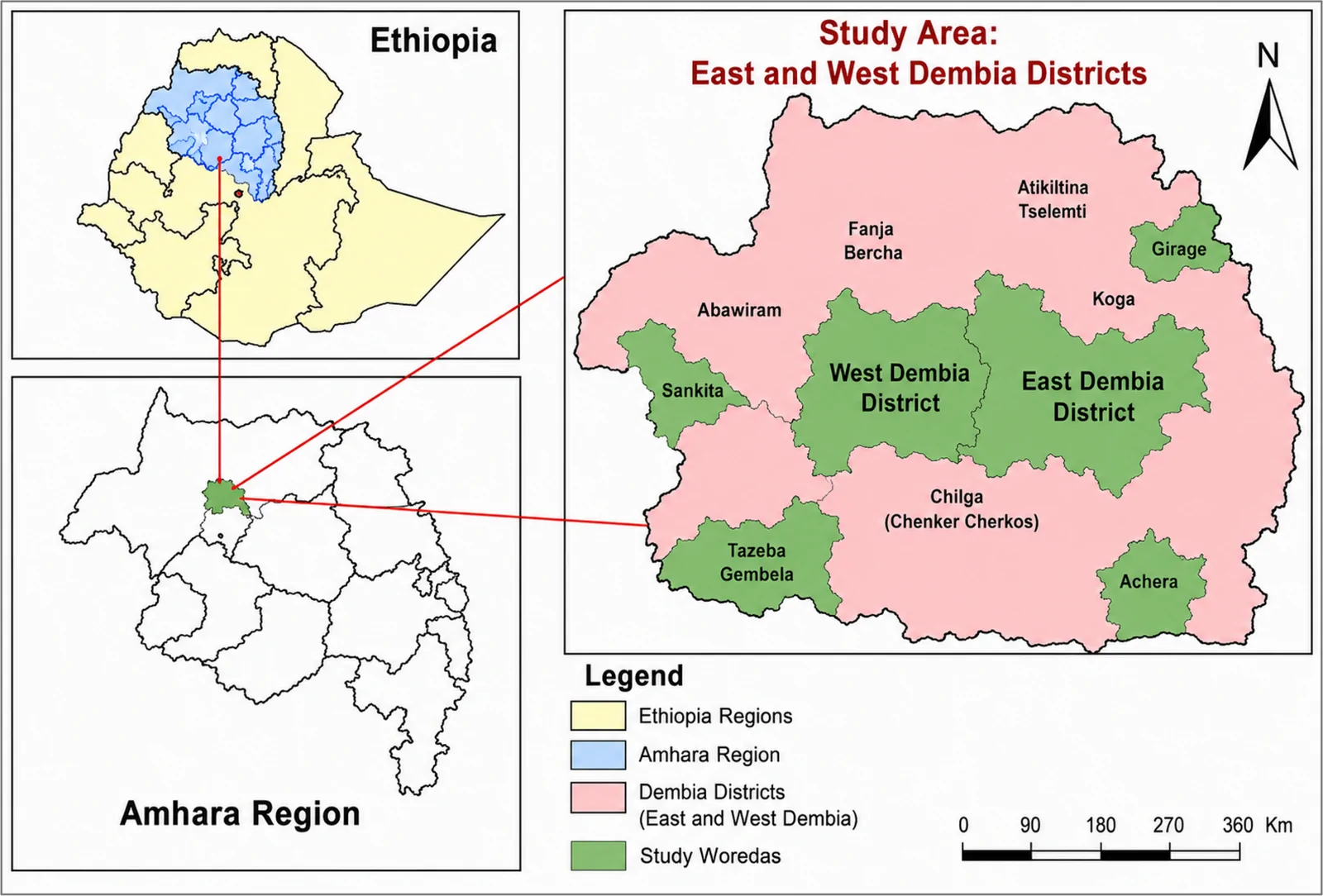
Map of the study area (ArcGIS 10.8)

The study was conducted from December 2024 to August 2025 in East and West Dembia districts (woredas), located in the Central Gondar Zone of northwest Ethiopia (Fig. 1). The districts are situated approximately 762 km northwest of Addis Ababa and about 245 km north of Bahir Dar. Geographically, the area lies at approximately 12°17′ N latitude and 37°27′ E longitude, with an average elevation of about 1,884 m above sea level. Elevation across the Dembia districts ranges from 1,500 to 2,600 m above sea level. The agro ecological zone is classified as midland (Woina Dega), with mean annual temperatures ranging from 11 °C to 32 °C and average annual rainfall between 995 and 1,175 mm (Tarekegn et al. 2021). East and West Dembia are bordered by Lake Tana to the south, Takusa to the southwest, Chilga to the west, Lay Armachiho to the north, and Gondar Zuria to the east. The selected peasant associations (PAs) in East Dembia included Chelo, Koladiba, Saleje, Gerarge, Sfankara, Wokerako, Fenja, Jangua, Atakelt, Robet, Adisga, and Guramba. In West Dembia, the sampled PAs were Abwuram, Achera, Cherkew, Tezeba, Chenker, Jenda, and Chuahit. Ethiopia is reported to own approximately 1.91 million horses, 6.75 million donkeys, and 0.35 million mules (Aliye et al. 2022).

### Study animals

The study included equines (horses, donkeys, and mules) of both sexes and aged over six months that had not been vaccinated against AHSV. Equines younger than six months were excluded to avoid potential interference from maternal antibodies, which may compromise serological test accuracy (Ahmed et al. 2018). This criterion is supported by Keith (2005), who reported that maternal antibodies in foals generally decline by six months of age. All animals were selected from East and West Dembia districts. According to 2024 reports from the respective District Livestock and Fishery Offices, the equine population in the study area comprised approximately 27,913 donkeys, 141 horses, and 89 mules.

### Study design

A cross-sectional study design was employed from December 2024 to August 2025 to estimate the seroprevalence of AHSV and to describe variations in seropositivity across selected explanatory variables among equines in the study area.

### Sample size determination

The sample size was calculated using the formula described by Thrusfield (2018), following epidemiological principles outlined by Bouyer (1991), with a 95% confidence level, 5% desired absolute precision, and an expected prevalence of 50%, as no prior data on AHSV antibody prevalence were available for the study areas.

$$\:n=\frac{{Z}^{2}\times\:Pexp\times\:(1-Pexp)}{{d}^{2}}$$

The sample size was calculated using the formula described by Thrusfield (2018), following epidemiological principles outlined by Bouyer (1991), with a 95% confidence level, 5% desired absolute precision, and an expected prevalence of 50%, as no prior data on AHSV antibody prevalence were available for the study areas.

Where: *n* = required sample size; *P*exp = expected prevalence (0.5); *d* = desired absolute precision (0.05); *Z* = Z-value for confidence level (1.96). Based on this formula, a total of 384 chickens were included in the study.

### Sampling technique

A five-stage sampling technique was employed. In the first stage, the Central Gondar Zone was purposively selected due to its large equine population and recurring AHS outbreaks. In the second stage, East and West Dembia districts were deliberately selected based on equine population density and previous reports of AHS occurrence, following approaches described by Ahmed et al. (2018) and Chuong et al. (2025). In the third stage, 20 peasant associations were selected based on accessibility and feasibility within the study period. Each PA was categorized according to equine population size as small (≤ 10 animals), medium (11–20 animals), or large (> 20 animals). In the fourth stage, households owning equines within each PA were identified using PA administrative records and served as the sampling frame. From this list, households were selected using simple random sampling. In the final stage, individual equines within selected households were sampled using a haphazard approach to approximate random selection, as individual animal registers were not available under the traditional extensive management system. The number and species of equines sampled from each PA were allocated proportionally to their respective population sizes, following methods described by Kim et al. (2024) and Bekele et al. (2024).

### Data collection

Approximately 5 mL of blood was collected aseptically from each selected animal via jugular venipuncture using sterile vacutainer systems. Animals were manually restrained to ensure safety and accuracy during sample collection. Blood samples were allowed to clot overnight at room temperature, after which serum was separated and transferred into labeled Eppendorf tubes. All serum samples were stored at − 20 °C until laboratory analysis. During sampling, relevant animal-level data were recorded, including species, age, sex, district and PA of origin, and movement history. Age was categorized as < 5 years and ≥ 5 years, following Molini et al. (2020), and was determined based on owner information and dentition patterns. A semi-structured questionnaire was administered to collect additional information on management practices (housing, grazing), health history, environmental factors, and equine movement patterns.

### Serological analysis

Serological testing was performed using a blocking enzyme-linked immunosorbent assay (bELISA) (INGEZIM AHSV COMPAC PLUS), following the manufacturer’s protocol. All laboratory analyses were conducted at the Animal Health Institute (AHI), Sebeta, Ethiopia. Optical density (OD) values were measured at 450 nm using an ELISA plate reader. The blocking percentage (BP) was calculated using the formula:

$$BP\;(\%)=\;\left[1-\frac{OD\;sample}{OD\;negative\;control}\right]\times100$$

Samples with BP values > 50% were considered positive, < 45% negative, and values between 45% and 50% were considered doubtful and retested, according to the manufacturer’s guidelines and WOAH recommendations.

### Data analysis and management

Data were entered into Microsoft Excel 2010 for cleaning and preliminary visualization and analyzed using Stata version 14. Seroprevalence was calculated as the proportion of seropositive animals among those tested, with 95% confidence intervals. Univariable logistic regression was used to assess associations between explanatory variables and AHSV seropositivity, and to estimate crude odds ratios (OR) with 95% confidence intervals. Data distribution across categories was examined prior to analysis. Some variables contained small numbers of observations in certain categories. Despite model convergence, this may have affected the precision of estimates. Multivariable logistic regression was not performed due to data sparsity, and results were interpreted as descriptive associations. Statistical significance was set at *p* < 0.05.

## Results

### Seroprevalence of African horse sickness virus

A total of 384 equines were sampled from East and West Dembia districts to determine the seroprevalence of African horse sickness virus (AHSV). Of these, 310 animals tested seropositive, giving an overall seroprevalence of 80.73% (95% CI: 76.42–84.55%).

In East Dembia district, 230 equines were examined, of which 187 were seropositive, corresponding to a seroprevalence of 81.30% (95% CI: 75.66–86.13%). At the peasant association (PA) level, seroprevalence varied from 66.67% in Saleje to 100% in Achera. Relatively high seroprevalence levels were observed in several PAs, including Atekilt (93.33%), Adisga (92.31%), Wokerako (90.91%), and Chelo (88.89%). Other PAs showed values ranging between approximately 72% and 82% (Table 1).

**Table 1:** Seroprevalence of African horse sickness virus (AHSV) among equines by peasant association (PA)

District	Peasant association	Sampled (*n*)	Positive (*n*)	Seroprevalence (%)	95% CI (Lower–Upper)
East Dembia	Kolladba	11	8	72.73	39.03–93.98
	Guranba	11	9	81.82	48.22–97.72
	Achera	11	11	100.00	71.51–100.00
	Adisga	13	12	92.31	63.97–98.81
	Atekilt	15	14	93.33	68.05–98.83
	Saleje	21	14	66.67	43.03–85.41
	Chelo	18	16	88.89	65.29–98.63
	Jangua	15	12	80.00	51.91–95.67
	Sfankara	25	19	76.00	54.87–90.64
	Fenja	26	21	80.77	60.65–93.45
	Robet	22	15	68.18	45.13–86.14
	Wokerako	22	20	90.91	70.84–98.88
	Geraregi	20	16	80.00	56.34–94.27
Subtotal	—	230	187	81.30	75.66–86.13
West Dembia	Chyeit	10	8	80.00	44.39–97.48
	Jenda	17	13	76.47	50.10–91.34
	Fendeka	20	15	75.00	50.90–91.34
	Tezba	22	20	90.91	70.84–98.88
	Abwuram	33	21	63.64	45.13–79.60
	Cherkaw	22	19	86.36	65.09–97.09
	Chenker	30	27	90.00	73.47–97.89
Subtotal	—	154	123	79.87	72.66–85.89
Total	—	384	310	80.73	76.42–84.55

Note: Seroprevalence estimates and 95% confidence intervals (CI) were calculated using exact binomial methods

In West Dembia district, 154 equines were sampled, of which 123 were seropositive, resulting in a seroprevalence of 79.87% (95% CI: 72.66–85.89%). At the PA level, seroprevalence ranged from 63.64% in Abwuram to 90.91% in Tezba, with most PAs showing values between 75% and 90% (Table 1).

### Risk factor analysis

Univariable logistic regression analysis was performed to explore associations between AHSV seropositivity and selected explanatory variables. No statistically significant associations were identified at the 5% significance level (*p* > 0.05). Although not statistically significant, some variables showed numerical differences in odds estimates. Equines from East Dembia had slightly higher odds of seropositivity compared to those from West Dembia (OR = 1.10; *p* = 0.727). Donkeys (OR = 3.53; *p* = 0.065) and horses (OR = 2.90; *p* = 0.208) had higher estimated odds compared to mules. Animals aged ≥ 5 years showed higher odds than younger equines (OR = 1.22; *p* = 0.443), while female equines had lower odds compared to males (OR = 0.67; *p* = 0.169). Equines under sheltered management appeared to have higher odds compared to mixed systems (OR = 1.78; *p* = 0.109). However, these differences should be interpreted with caution as they were not statistically significant and may reflect underlying data variability. No apparent differences were observed for clinical signs, feed source, movement frequency, or proximity to water bodies (Table 2).

**Table 2:** Univariable logistic regression analysis of factors associated with AHSV seropositivity in equines

Risk factor	Category	Tested (*n*)	Positive (*n*)	Prevalence (%)	Odds Ratio (OR)	95% CI	*p*- value
District	East Dembia	230	187	81.30	1.10	0.67–1.83	0.727
	West Dembia	154	123	79.87	Ref.	—	—
Species	Donkey	352	287	81.53	3.53	0.92–13.52	0.065
	Horse	23	18	78.26	2.90	0.56–14.94	0.208
	Mule	9	5	55.56	Ref.	—	—
Age (years)	≥ 5	233	191	81.97	1.22	0.73–2.05	0.443
	< 5	151	110	72.85	Ref.	—	—
Sex	Female	254	198	77.95	0.67	0.38–1.17	0.169
	Male	130	112	86.15	Ref.	—	—
Housing	Open	105	84	80.00	1.47	0.68–3.18	0.328
	Sheltered	227	188	82.82	1.78	0.88–3.59	0.109
	Both	52	38	73.08	Ref.	—	—
Feed source	Local market	40	33	82.50	1.55	0.49–4.92	0.748
	Both	18	15	83.33	1.22	0.34–4.34	0.757
	Farm produced	326	262	80.37	Ref.	—	—
Movement	Weekly	74	64	86.49	1.68	0.81–3.47	0.162
	Rarely	31	25	80.65	1.09	0.43–2.76	0.852
	Daily	279	221	79.21	Ref.	—	—
Water body	No	169	138	81.66	1.11	0.67–1.84	0.683
	Yes	215	172	80.00	Ref.	—	—

Note: Odds ratios (OR) with 95% confidence intervals (CI) were obtained from univariable logistic regression models. Reference categories are indicated (Ref.)

## Discussion

AHSV remains one of the most important vector-borne viral pathogens affecting equines in sub-Saharan Africa. Understanding its epidemiology in endemic settings is essential for informing effective prevention and control strategies. The present study provides updated evidence on the seroepidemiology of AHSV in equines from East and West Dembia districts of the Central Gondar Zone, northwest Ethiopia, demonstrating widespread exposure to the virus within the study area.

The overall seroprevalence of AHSV observed in this study (80.73%) is higher than earlier reports from Ethiopia, where seroprevalence values ranging from 14.23% to 46.2% have been documented in areas such as Arsi and Bale zones, Jimma zone, and the central highlands (Tilahun et al 2012; Bitew et al. 2011; Azeez 2017). Kassa (2006) similarly reported a lower overall prevalence of 23%, with variation among equine species. These differences may be attributed to variation in ecological conditions, vector abundance, sampling periods, diagnostic methods, and management practices. In particular, the midland agro-ecological conditions of the study area may favor sustained circulation of Culicoides vectors, contributing to high levels of exposure. However, caution is required when comparing results across studies due to differences in methodology and study design (Chávez-Tinoco et al. 2025).

Species-specific seroprevalence estimates showed slightly higher seropositivity in donkeys compared with horses and mules; however, these differences were not statistically significant and should be interpreted as descriptive observations rather than indicative of true differences in risk. Donkeys are known to develop predominantly mild or subclinical infections, often referred to as horse sickness fever, which may go undetected during outbreaks (Mellor and Hamblin 2004; Maclachlan and Guthrie 2010; WOAH 2025). Because of their lower mortality and prolonged survival following infection, donkeys may remain available for repeated vector exposure and can serve as useful sentinel animals in endemic settings, as previously suggested in Ethiopia and other regions (Wilson et al. 2008; Oura et al. 2012; Thompson et al. 2012; Tinarwo et al. 2025).

The high seroprevalence observed across both East and West Dembia districts suggests widespread exposure to AHSV throughout the study area rather than localized transmission. The districts are characterized by environmental conditions conducive to Culicoides breeding and survival, including warm temperatures, seasonal rainfall, high humidity, and proximity to Lake Tana, rivers, irrigation systems, and shaded organic-rich environments (Lefèvre and Calvez 1986; Meiswinkel 2004; Rodríguez et al. 2020). Similar patterns have been reported in other endemic regions, where ecological suitability supports continuous vector activity and viral circulation.

Comparable levels of high seroprevalence have been reported in studies from Sudan and Nigeria, indicating widespread exposure and long-term circulation of AHSV in multiple sub-Saharan African settings (Karamalla et al. 2018; Chinyere et al. 2025). These observations suggest consistent epidemiological patterns across the region and highlight the importance of sustained surveillance. Ongoing monitoring is particularly relevant in the context of environmental and climatic variability, which may influence vector distribution and disease dynamics (Haji Ende et al. 2013; Negessu and Worku 2020; Acosta et al. 2025).

Although none of the evaluated risk factors were statistically associated with seropositivity, some variation in seroprevalence was observed across species, age groups, sex, and management categories. Older equines showed higher seropositivity, which may reflect cumulative exposure over time, a pattern commonly observed in endemic vector-borne infections (Karamalla et al. 2018). Similarly, differences observed across housing systems may reflect varying degrees of exposure to vector habitats, as Culicoides species are known to exploit both outdoor and sheltered microenvironments (Meiswinkel et al. 2004; Zientara et al. 2015; Assefa et al. 2022). However, these patterns were not statistically significant and should be interpreted cautiously

Importantly, the absence of statistically significant associations may be related to limitations in sample distribution across categories and the presence of sparse data in some groups, which can reduce statistical power and the precision of estimates. This is further reflected by the relatively wide confidence intervals observed for several variables. As a result, the findings should be considered as indicative of general exposure patterns rather than definitive evidence of specific risk factors.

## Limitations

This study has several limitations. Serological testing does not distinguish between past exposure and active infection, nor identify circulating AHSV serotypes. Sparse data in some categories resulted in wide confidence intervals and limited the analysis to univariable logistic regression, preventing assessment of confounding. Additionally, the cross-sectional design does not allow causal inference. Despite these limitations, the study provides important baseline data on AHSV exposure in a high-risk, under-studied area.

## Conclusion 

This study revealed a high seroprevalence of African horse sickness virus (AHSV) among equines in East and West Dembia, indicating widespread exposure in the study area. No statistically significant associations were identified between seropositivity and the evaluated factors, although some variations were observed across categories. These findings underscore the continued importance of AHS as a threat to equine health and livelihoods. Further studies using larger sample sizes, multivariable approaches, and integrated diagnostic methods are recommended. Strengthening vaccination, improving management practices, and enhancing vector control remain essential for mitigating disease impact.

## Data Availability

Data available upon request from the corresponding author.

## Abbreviations

AHSV: African horse sickness virus
OR: odds ratio
CI: confidence interval
Ref.: reference category

## Declarations

### Animal welfare statement

This study was approved by the Ethics Research Review Committee of the College of Veterinary Medicine and Animal Sciences, University of Gondar (Ref. No. CVMASc/UoG/RERC/008/2025, December 15, 2024). All procedures were conducted in accordance with relevant national and international guidelines for animal research, including the ARRIVE guidelines. Prior to data collection, permission was obtained from the University of Gondar. Blood sampling was carried out with minimal animal discomfort following standard veterinary procedures. Informed oral consent was obtained from all animal owners after explaining the purpose of the study, confidentiality, and potential benefits and risks.
