# Correction to: Seroprevalence and risk factors of African horse sickness in equines in central Gondar, Ethiopia

**DOI:** 10.1007/s11259-026-11406-x

**Published:** 2026-07-23

**Authors:** Abebech Atinafu Yehulashet, Fentahun Wondmnew, Anmaw Shite Abat, Enyiew Alemnew Alamerew, Mastewal Birhan, Firdyawukal Abuhay Tafere, Birhan Anagaw Malede, Andebet Sechu, Amsalu Chanie Mersha, Elias Melkamu Tsehay, Abraham Belete Temesgen

**Affiliations:** 1 Department of Veterinary Pharmacy, College of Veterinary Medicine and Animal Sciences, University of Gondar, P.O. Box: 196, Gondar, Ethiopia; 2 Department of Veterinary Pathobiology, College of Veterinary Medicine and Animal Sciences, University of Gondar, Gondar, Ethiopia; 3 Amhara Agricultural Research Institute, Debre Birhan Agricultural Research Centre, Debre Birhan, Ethiopia; 4 Department of Serology, Animal Health Institute, Sebeta, Ethiopia; 5 Department of Veterinary Epidemiology and Public Health, College of Veterinary Medicine and Animal Sciences, University of Gondar, Gondar, Ethiopia

**Correction to: Veterinary Research Communications (2026) 50:445**

**https://doi.org/10.1007/s11259-026-11388-w**

In this article, the author’s name Enyiew Alemnew Alamerew was incorrectly written as Enyiew Alemnew Alamere. The affiliation details for Enyiew Alemnew Alamerew were also incorrectly given as Debre Birhan Agricultural Research Centre, Debre Birhan, Ethiopia ' but should have been 'Amhara Agricultural Research Institute, Debre Birhan Agricultural Research Centre, Debre Birhan, Ethiopia'.

The original article has been corrected.

